# Monitoring Astrocytic Ca^2+^ Activity in Freely Behaving Mice

**DOI:** 10.3389/fncel.2020.603095

**Published:** 2020-12-03

**Authors:** Han Qin, Wenjing He, Chuanyan Yang, Jin Li, Tingliang Jian, Shanshan Liang, Tunan Chen, Hua Feng, Xiaowei Chen, Xiang Liao, Kuan Zhang

**Affiliations:** ^1^Brain Research Center and State Key Laboratory of Trauma, Burns, and Combined Injury, Third Military Medical University, Chongqing, China; ^2^Center for Neurointelligence, School of Medicine, Chongqing University, Chongqing, China; ^3^Department of Neurosurgery and Key Laboratory of Neurotrauma, Southwest Hospital, Third Military Medical University, Chongqing, China

**Keywords:** optic fiber, astrocytes, Ca^2+^ signals, genetically encoded Ca^2+^ indicators, freely behaving mice, cortex, hippocampus

## Abstract

Monitoring astrocytic Ca^2+^ activity is essential to understand the physiological and pathological roles of astrocytes in the brain. However, previous commonly used methods for studying astrocytic Ca^2+^ activities can be applied in only anesthetized or head-fixed animals, which significantly affects *in vivo* astrocytic Ca^2+^ dynamics. In the current study, we combined optic fiber recordings with genetically encoded Ca^2+^ indicators (GECIs) to monitor astrocytic activity in freely behaving mice. This approach enabled selective and reliable measurement of astrocytic Ca^2+^ activity, which was verified by the astrocyte-specific labeling of GECIs and few movement artifacts. Additionally, astrocytic Ca^2+^ activities induced by locomotion or footshock were stably recorded in the cortices and hippocampi of freely behaving mice. Furthermore, this method allowed for the longitudinal study of astrocytic activities over several weeks. This work provides a powerful approach to record astrocytic activity selectively, stably, and chronically in freely behaving mice.

## Introduction

Brain astrocytes are abundant glial cells that tile the central nervous system (Khakh and Sofroniew, 2015). These ubiquitous cells respond to neuronal activity with increased intracellular Ca^2+^ concentrations *via* the activation of various receptors and then release gliotransmitters, which in turn act on neurons (Haydon and Nedergaard, 2014; Bazargani and Attwell, 2016; Zhang and Chen, 2017). By such direct bidirectional interactions, astrocytic Ca^2+^ transients serve as significant markers of astrocyte integration into neuronal networks (Halassa and Haydon, 2010; Santello et al., 2019). Increasing evidence indicates that astrocytic Ca^2+^ transients are behaviorally relevant (Shigetomi et al., 2016) and required for cognitive functions (Halassa and Haydon, 2010; Santello et al., 2019). Also, the regulation of Ca^2+^ dynamics is dysfunctional in neuronal diseases (Kuchibhotla et al., 2009; Verkhratsky et al., 2017). Hence, recording these transients in freely behaving mice is essential to elucidate the role of astrocytes in physiological and pathological processes and diseases, such as learning and memory (Adamsky et al., 2018), hyperactivity (Nagai et al., 2019), depression (Wang et al., 2017) and Alzheimer’s disease (Verkhratsky et al., 2017).

Astrocytic Ca^2+^ signals in the brain have been investigated *in vivo* for almost 20 years (Zhang and Chen, 2017). Most of these studies were conducted with two-photon microscopy (Hirase et al., 2004; Bekar et al., 2008; Nimmerjahn et al., 2009; Ding et al., 2013; Paukert et al., 2014; Oe et al., 2020) and camera-based large-field imaging (Ghosh et al., 2013; Daniel et al., 2015) in anesthetized or head-fixed awake mice. However, anesthesia and restraint-induced stress affect astrocytic Ca^2+^ dynamics *in vivo* (Schummers et al., 2008; Nimmerjahn et al., 2009; Thrane et al., 2012; Oliveira et al., 2015; Oe et al., 2020). Furthermore, these techniques were largely limited to the imaging of upper cortical layers (Hirase et al., 2004; Bekar et al., 2008; Ghosh et al., 2013; Daniel et al., 2015; Oe et al., 2020). Complementary to these limitations, the optic fiber-based Ca^2+^ recording approach can be applied to obtain deep-tissue measurements in freely behaving animals (Adelsberger et al., 2014; Zhang et al., 2017; Qin et al., 2018; Yao et al., 2018, 2019; Li et al., 2019) and to record the population activities of cell bodies, axon terminals and dendrites in neurons(Grienberger et al., 2012; Stroh et al., 2013; Qin et al., 2018, 2019; Yao et al., 2018). This technique has also been applied in astrocytic Ca^2+^ recording in head-fixed mice and stably displays cortical astrocytic Ca^2+^ changes (Schulz et al., 2012; Paukert et al., 2014). However, until now, no studies have demonstrated *in vivo* forms of astrocytic activities in freely behaving animals.

Genetically encoded Ca^2+^ indicators (GECIs) comprise a fluorescent protein and Ca^2+^-binding motif and display increased fluorescence in the presence of Ca^2+^ (Hires et al., 2008; Broussard et al., 2014). They overcome the limitations of the commonly used organic Ca^2+^ indicators and display many advanced characteristics, such as cell-specific targeting(Chai et al., 2017; Yu et al., 2018; Nagai et al., 2019), a high signal-to-noise ratio (Shigetomi et al., 2013), little bleaching (Shigetomi et al., 2013) and stable expression for weeks (Paukert et al., 2014; Srinivasan et al., 2015). Hence, GECIs have recently become a strong tool for *in vivo* astrocytic Ca^2+^ recording (Shigetomi et al., 2013, 2016; Yu et al., 2020). Therefore, the combination of GECIs and optic fiber-based Ca^2+^ recording provides an ideal approach to record astrocytic Ca^2+^ transients in freely behaving mice.

In this study, we combined optic fiber-based Ca^2+^ recording with GECIs and monitored locomotion and footshock-evoked astrocytic activities in freely behaving mice. Furthermore, we chronically recorded astrocytic Ca^2+^ transients in the brains of freely behaving mice for several weeks *via* this method. The results indicate that optic fiber-based Ca^2+^ recording combined with GECIs is an ideal approach to dissect the functions of astrocytes in physiological and pathological brain processes.

## Materials and Methods

### Animals

C57BL/6J mice (3–4 months old, male) were used in all experiments and purchased from the Laboratory Animal Center at the Third Military Medical University. Mice were housed in groups under a 12-h light/dark cycle with free access to food and water; mice implanted with optic fibers were individually housed. All experimental procedures were performed according to institutional animal welfare guidelines and were approved by the Third Military Medical University Animal Care and Use Committee.

### Surgery and Adeno-Associated Virus (AAV) *In vivo* Microinjections

The mice were anesthetized with 1–2% isoflurane in oxygen and then placed in a stereotactic head frame (RWD Technology Company Limited, China) with a heating pad (37.5–38°C) underneath. A small vertical incision was made in the skin, and a craniotomy (0.5 mm × 0.5 mm) was performed with a dental drill. AAV5-GfaABC_1_D-cyto-GCaMP6f- SV40 (Cat# AV-5-52925, UPenn vector core; Shigetomi et al., 2016; Adamsky et al., 2018; Nagai et al., 2019; Yu et al., 2020), which is a genetically encoded Ca^2+^ indicator driven by the astrocyte-specific *GfaABC_1_D* promoter (a shorter 681 bp GFAP promoter; Shigetomi et al., 2016; Nagai et al., 2019; Yu et al., 2020), was injected at a volume of 500 nl/site without dilution, while AAV5-GfaABC_1_D-PI-Lck-GFP-SV40 (Cat# AV-5-PV2369, UPenn vector core) was injected as a control. The coordinates for the auditory cortex were as follows: anteroposterior (AP): −3.1 mm; mediolateral (ML): ±3.8 mm; and dorsoventral (DV): −1.25 mm with a 20 degrees slope below the dura. These coordinates for the hippocampal CA1 region were as follows: AP: −2.18 mm; ML: ±1.60; and DV: 1.2 mm from the dura. The injection coordinates were based on the Paxinos and Franklin (2004). Depending on the spread of the reagent injected, 1–3 injection sites were utilized at the DV axis to achieve sufficient coverage of the desired region. A glass micropipette with a tip diameter of 10–20 μm was inserted to infuse the virus. After each injection, the micropipette was held in place for 5–10 min before being slowly retracted. The scalp incision was closed with tissue glue (3M Animal Care Products, Vetbond), and postinjection analgesics were provided for 3 days to facilitate recovery. The experiments were performed during the time window of 4–6 weeks postinjection.

### Optic Fiber Setup

A custom-built fiber photometry setup (Figure 2A) was used for recording the astrocytic Ca^2+^ transients throughout the entire study (Zhang et al., 2017; Qin et al., 2018; Yao et al., 2019). The fluorescent genetically encoded Ca^2+^ indicator (GCaMP6f) was excited by light (wavelength of 488 nm) delivered by a laser (OBIS 488 LX-50 mW, Coherent, Santa Clara, CA, USA), and the beam intensity of the laser was accurately adjusted by the current. Then, the beam was deflected by a dichroic mirror (Di02-R488, Semrock, West Henrietta, NY, USA) and focused on the end of an optic fiber (200 μm diameter, NA 0.48, Doric lenses, Quebec City, QC, Canada) through a collimator. The beam intensity at the tip of the optic fiber was approximately 0.22 mW/mm^2^. The emitted GCaMP6f fluorescence (green) was guided back using the same fiber and then separated from excitation blue light by the same dichroic mirror and a bandpass emission filter (FF01-535/22, Semrock, West Henrietta, NY, USA). Finally, the GCaMP6f fluorescence emission was detected by an avalanche photodiode (Si APD, S2382, Hamamatsu Photonics K.K., Japan). The digitization of the collected GCaMP6f fluorescence signals was achieved by a multifunction I/O device (USB-6002, National Instruments, Austin, TX, USA) with a sampling frequency of 2000 Hz. Data were acquired by customized software written on the LabVIEW platform (National Instrument, Austin, TX, USA).

**Figure 1:**
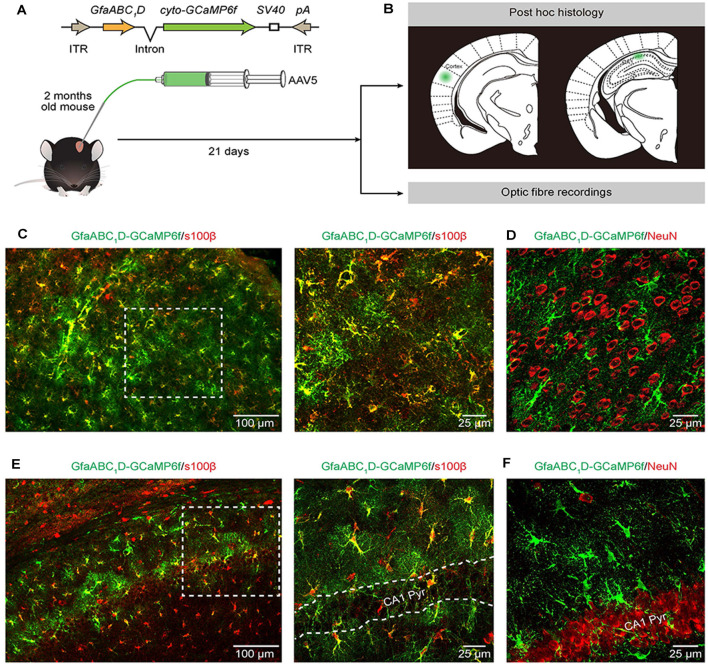
Expression of AAV5-GfaABC_1_D-cyto-GCaMP6f-SV40 throughout astrocytes in the cortex and hippocampus. **(A)** The top cartoon shows the design of AAV5 constructs used to deliver cyto-GCaMP6f; the bottom schematic illustrates the protocol for AAV5 microinjections and the following experimental approaches **(B)**. **(B)** *Post hoc* analyses (top) to confirm the sites of cyto-GCaMP6f expression (the green regions) in the cortex (left) and hippocampal CA1 area (right). Optic fiber recordings of astrocytic Ca^2+^ transients after virus injection (bottom).** (C)** Representative image showing GCaMP6f (green) and s100β (red) immunostaining in the cortices of mice that received AAV5-GfaABC_1_D-cyto-GCaMP6f-SV40 (left). High-magnification image (right) showing immunostaining of GCaMP6f (green) and s100β (red) as indicated by the white dashed box in the left panel. **(D)** Representative image showing GCaMP6f (green) and NeuN (red) immunostaining in the cortices of mice after virus injection. **(E)** Representative image showing GCaMP6f (green) and s100β (red) immunostaining in the hippocampal CA1 area of mice that received AAV5-GfaABC_1_D-cyto-GCaMP6f-SV40 (left). High-magnification image (right) showing immunostaining of GCaMP6f (green) and s100β (red) as indicated by the white dashed box in the left panel. The outlined area in the right panel is the CA1 pyramidal layer (CA1 Pyr). **(F)** Representative image showing GCaMP6f (green) and NeuN (red) immunostaining in the hippocampal CA1 area of mice after virus injection.

**Figure 2:**
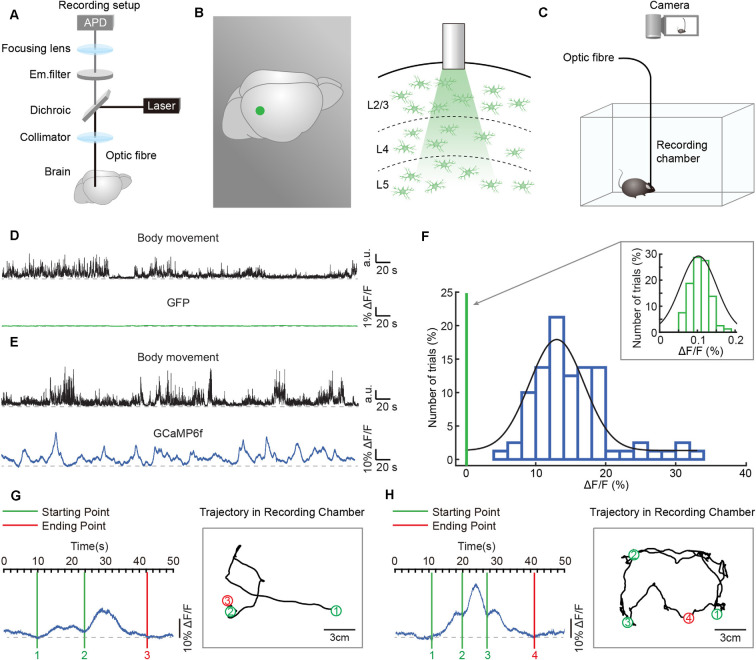
Simultaneous recordings of astrocytic Ca^2+^ transients and body movements during a freely behaving state. **(A)** Scheme of the optic fiber recording setup. **(B)** Schematic showing the location (left panel: green point) and the tip of the optic fiber (diameter: 200 μm) implanted in layer 2/3 of the auditory cortex (right panel).** (C)** Schematic of the recording setup in which astrocytic Ca^2+^ transients and behavior were recorded simultaneously. **(D)** Example showing body movements (black traces) and simultaneously recorded fluorescence (green trace) of the AAV5-GfaABC_1_D-PI-Lck-GFP-SV40-injected group. **(E)** Example showing body movements (black traces) and simultaneously recorded astrocytic Ca^2+^ transients (blue trace) of the AAV5-GfaABC_1_D-cyto-GCaMP6f-SV40-injected group. **(F)** Distributions of the amplitudes of GCaMP6f fluorescence (blue histogram) and GFP fluorescence (upper right, green histogram, which is expanded from the green line on the left of the blue histogram). Both histograms fit Gaussian distributions. The mean values were 15.27% ΔF/F in the GCaMP6f group and 0.11% ΔF/F in the GFP group. **(G,H)** Examples showing the trial by trial correlation between the starting/ending points of astrocytic Ca^2+^ transients (left) and specific locations of freely behaving mice (right). Circles (green/red) and black lines indicate the positions where astrocytic Ca^2+^ transients start/end and the trajectories of the mice, respectively. The rectangular gray boxes indicate the recording chamber. Data is from two different mice.

### Optic Fiber Recordings in Freely Behaving Mice

Mice were anesthetized with 1–1.5% isoflurane and then placed in a stereotactic head frame (RWD Technology Company Limited, China) with a heating pad (37.5–38°C) underneath.

A small vertical incision was made in the skin, and a craniotomy (1 mm × 1 mm) was performed using a dental drill above the previous virus injection sites in the auditory cortex or hippocampus. An optic fiber (200 μm diameter, NA 0.48, Doric lenses, Quebec City, QC, Canada) was glued into a short cannula (ID. 0.51 mm, OD. 0.82 mm), inserted through the craniotomy, and advanced slowly to 50 μm above the previous virus injection sites. The optic fiber was fixed to the mouse skull using dental cement. After implantation of the optic fibers, mice were allowed to recover for 3–5 days before astrocytic Ca^2+^ transient recording. Changes in fiber curvature were checked every 6 h to avoid breakage in freely behaving mice. The mice were put into a white rectangular recording chamber in which they could move freely. Every mouse was continuously recorded for 1 h for locomotion-evoked Ca^2+^ transients or 10 trials for footshock-induced astrocytic Ca^2+^ transients by customized acquisition software based on the LabVIEW platform (National Instrument, Austin, TX, USA). A camera (Aigo AHD-X9, China) was used to simultaneously record mouse behavior at 30 Hz under a spatial resolution of 1,280 × 720 pixels.

### Histology and Confocal Imaging

Mice were transcardially perfused with 4% paraformaldehyde after optic fiber recording. The brains were removed, fixed overnight in 4% paraformaldehyde prepared with 15% sucrose, and cryoprotected in 30% sucrose. Coronal brain slices (40 μm thick) were obtained and stained as described (Zhang et al., 2016; Wang et al., 2018). Briefly, brain slices were blocked at room temperature for 30 min in 10% normal goat serum, 1% bovine serum albumin, and 0.3% Triton X-100 in PBS and incubated with primary antibodies overnight at 4°C (rabbit anti-NeuN, 1:500, Abcam, 177487; rabbit anti-S100β, 1:500, SYSY, 287003; chicken anti-GFP, 1:500, Abcam, ab13970). Slices were rinsed in PBS, followed by incubation with secondary antibodies directed against immunoglobulins of the appropriate species coupled to Alexa 594 and 488 (Invitrogen, 1:500). Images were obtained with a Leica SP5 confocal microscope equipped with standard filter sets and oil immersion objectives (60×/1.42 and 20×/0.85).

### Data Analysis

Astrocytic Ca^2+^ transients were acquired at the sampling frequency of 2,000 Hz and low-pass filtered by the Butterworth filter. For the optic fiber recordings in freely behaving mice, the relative fluorescence changes (Δf/f = (f − f_baseline_)/f_baseline_) were calculated for Ca^2+^ transients, where f_baseline_ was the baseline fluorescence level taken during the current test recording period (Zhang et al., 2017; Yao et al., 2018). A template-matching algorithm was used, taking into account the properties of the rise and decay times of the Ca^2+^ signals, to automatically detect the astrocytic Ca^2+^ transients (Zhang et al., 2017). A Ca^2+^ transient was regarded as a signal if its amplitude was three times larger than the standard deviation of the noise band (Zhang et al., 2017; Qin et al., 2018; Yao et al., 2018). We converted the image frames into binary format to obtain the mouse shape according to the image intensities and calculated the mouse movement by the change in video clip image relative to body size. Traces of astrocytic Ca^2+^ transients were analyzed around the onset of locomotion/footshock or at randomly selected times within the same assay (shuffled data) as a control. The data analysis software was custom-written in MATLAB 2018b (MathWorks, USA).

### Statistical Analysis

Data are expressed as the mean ± SEM. Investigators were blinded to group allocation during data analysis. When comparing three or more groups, we use the Kruskal–Wallis test; n.s., no significant difference.

## Results

### Specific and Efficient Expression of GECIs in Astrocytes

GCaMP6f GECI was employed because previous *in vivo* characterization studies have demonstrated its higher sensitivity compared to that of commonly used synthetic Ca^2+^ dyes (Chen et al., 2013) and revealed extensive Ca^2+^ fluctuations within astrocytes (Shigetomi et al., 2013; Srinivasan et al., 2015; Yu et al., 2020). We used serotype 5 of AAV (Ortinski et al., 2010) and the minimal astrocyte-specific *GfaABC_1_D* promoter (Xie et al., 2010; Shigetomi et al., 2016; Nagai et al., 2019; Yu et al., 2020) to selectively express cytosolic GCaMP6f (cyto-GCaMP6f) within astrocytes located in the cortex and hippocampus of the adult mouse (Figure 1A). Twenty-one days after the microinjection of AAV5, the sites of cyto-GCaMP6f expression were confirmed by *post hoc* histology. Then, optic fiber recordings were applied according to the sites of cyto-GCaMP6f expression in the following experiments (Figure 1B).

This result indicated that microinjection of AAV5 *in vivo* resulted in reliable, robust, and mosaic expression of GCaMP6f within astrocytes in the cortices and hippocampal CA1 area of adult mice (Figures 1C–F). Based on immunohistochemical analysis, GCaMP6f expression was almost colocalized with the astrocyte marker s100β (Figures 1C,E) and not detected within neurons (Figures 1D,F) in either the cortex or hippocampus. The statistical results further indicated that the GCaMP6f expression specificity was 94.8 ± 2.9% in the cortex and 85.8 ± 2.4% in the hippocampus (Table 1), while its expression efficiency was 94.2 ± 2.7% in the cortex and 90.4 ± 3.5% in the hippocampus (Table 1), demonstrating that the combination of AAV5 and the *GfaABC_1_D* promoter selectively and efficiently targets astrocytes.

**Table 1:** Specificity and efficiency of AAV5-GfaABC_1_D-cyto-GCaMP6f labelling.

Specificity (%/section)	Cortex (%)	Hippocampus (%)
%S100β^+^ GCaMP6f^+^/GCaMP6f^+^	94.8 ± 2.9 (*n* = 5 mice)	85.8 ± 2.4 (*n* = 5 mice)
**Efficiency (%/section)**	**Cortex (%)**	**Hippocampus (%)**
%S100β^+^ GCaMP6f^+^/S100β^+^	94.2 ± 2.7 (*n* = 5 mice)	90.4 ± 3.5 (*n* = 5 mice)

### Astrocytic Ca^2+^ Transient Recording in Freely Behaving Mice

We applied the optic fiber-based approach to record astrocytic Ca^2+^ transients in freely behaving mice. This fiber recording device is described in our previous work (Zhang et al., 2017; Yao et al., 2018, 2019) and allows the excitation of GCaMP6f and the collection of emitted light (Figure 2A). An optic fiber with a diameter of 200 μm was implanted above GCaMP6f-positive astrocytes (Figure 2B) 3 weeks after virus injection (AAV5-GfaABC_1_D-cyto-GCaMP6f, Figure 1A). Each mouse was placed in a white, opaque, rectangular chamber 24 h after optic fiber implantation, and its behavior was recorded using a camera placed above the chamber (Figure 2C).

To visualize the astrocytic Ca^2+^ transients recorded by optic fibers in freely behaving mice without undue levels of movement artifacts, we compared the optic fiber recording data between the GFP-injected control group (AAV5-GfaABC_1_D-PI-Lck-GFP-SV40) and the GCaMP6f-injected group (AAV5-GfaABC_1_D-cyto-GCaMP6f-SV40). A representative example revealed that movement-related transients were not observed in the GFP-injected control group (Figure 2D), while astrocytic Ca^2+^ transients were obvious and stable in the GCaMP6f-injected group during free movement (Figure 2E). The statistical results further confirmed that the mean value of optic fiber-recorded transients was 15.27% in the GCaMP6f-injected group (Figure 2F, blue histogram) and only 0.11% in the GFP-injected control group (Figure 2F, upper right, green histogram). Also, the distributions of the amplitudes of GCaMP6f (Figure 2F, blue histogram) and GFP fluorescence (Figure 2F, upper right, green histogram) indicated that the transients recorded in the GCaMP6f group were several 100 times greater than those in the GFP group (Figure 2F). These experiments showed that the astrocytic Ca^2+^ transients recorded by optic fibers in freely behaving mice were without movement artifacts.

To investigate the role of astrocytic Ca^2+^ transients in cognition functions, such as exploring and spatial memory, the astrocytic activities, and the real-time locations of mice have to be recorded at the same time. In the present study, we acquire the simultaneous recordings of astrocytic Ca^2+^ transients and the motion of freely behaving mice (Figure 2C). The specific locations where the astrocytic Ca^2+^ transients start/end can be clearly labeled in the trajectories of mice according to the simultaneously recorded videos (Figures 2G,H), which cannot be easily done by the traditional imaging methods, such as *in vivo* two-photon imaging.

### Locomotion-Induced Astrocytic Ca^2+^ Transients in the Cortices and Hippocampi of Freely Behaving Mice

**Figure 3:**
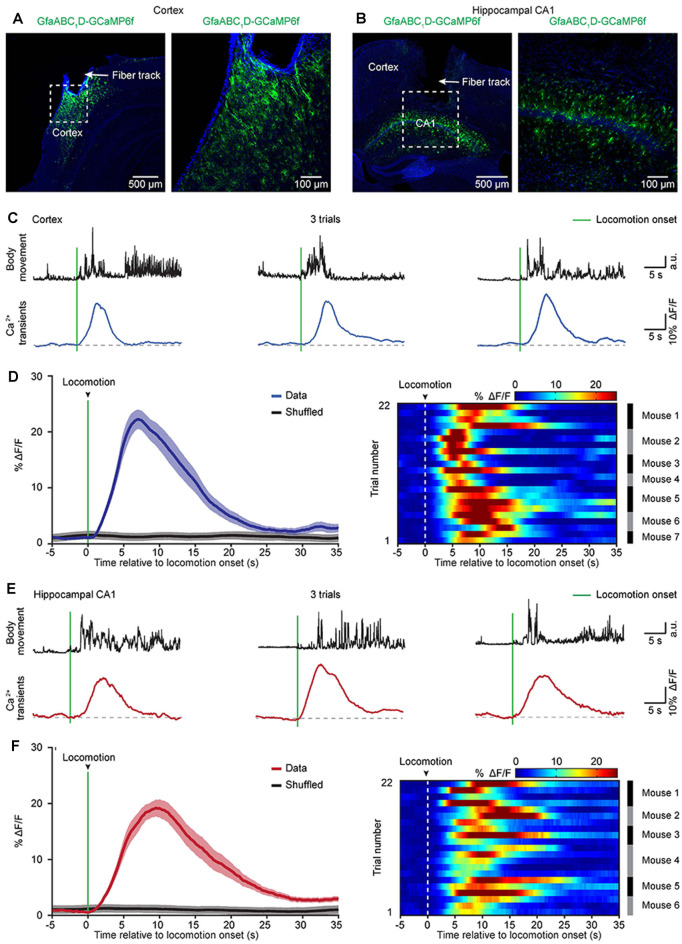
Astrocytic Ca^2+^ transients induced by locomotion in the cortices and hippocampi of freely behaving mice. **(A,B)** Representative *post hoc* images showing AAV5-GfaABC_1_D-cyto-GCaMP6f-labeled astrocytes below the fiber tracks (arrowhead) in the cortex **(A)** and hippocampal CA1 area **(B)**. The right panels are high-magnification images as indicated by the white dashed box in the left panels **(A,B)**.** (C)** Representative trials showing body movements (top, black traces) and astrocytic Ca^2+^ transients (bottom, blue traces) simultaneously recorded in the cortex. The green bars indicate locomotion onsets. **(D)** Left, the average (blue trace) of astrocytic Ca^2+^ transients aligned to locomotion onset (green bar) in the cortices. The average trace was from 22 trials (seven mice). The shaded area represents SEM. Right, color-coded intensities of astrocytic Ca^2+^ transients from different trials in the cortices. The mice which the trials of astrocytic Ca^2+^ transients are from are indicated in the right black and gray column. **(E)** Representative trials showing body movements (top, black traces) and astrocytic Ca^2+^ transients simultaneously (bottom, red traces) recorded in the hippocampus. The green bars indicate the locomotion onsets. **(F)** Left panel, the average (red trace) of astrocytic Ca^2+^ transients aligned to locomotion onset (green bar) in the hippocampi. The average trace was from 22 trials (six mice). The shaded area represents SEM. Right panel: color-coded intensities of astrocytic Ca^2+^ transients from different trials in the hippocampi. The mice which the trials of astrocytic Ca^2+^ transients are from are indicated in the right black and gray column.

Previous two-photon imaging studies in head-strained mice have shown that locomotion triggers widespread astrocytic activation in multiple brain regions (Nimmerjahn et al., 2009; Paukert et al., 2014). Next, we aimed to determine whether locomotion-induced astrocytic Ca^2+^ transients could be recorded in the cortical and subcortical areas of freely behaving mice by optic fibers. To avoid Ca^2+^ signal contamination in nearby brain areas, we recorded astrocytic activities in two brain regions that are separate from each other (cortical area: auditory cortex, subcortical area: hippocampus). Three weeks after virus injection, an optic fiber with a diameter of 200 μm was implanted above GCaMP6f-positive astrocytes in the auditory cortex (Figure 3A) or the hippocampal CA1 area (Figure 3B), and recordings were performed at least 24 h after fiber implantation. In this study, we simultaneously recorded both astrocytic Ca^2+^ transients and body movements during the freely behaving state (Figure 2C). Mouse body movement was recorded with a camera placed above the recording chamber (Figure 2C) and is shown as black traces (Figures 3C,E). We found reliable locomotion-induced astrocytic Ca^2+^ transients in both the cortex (Figure 3C) and the hippocampus (Figure 3E). Further analysis indicated that most of the astrocytic Ca^2+^ transients started approximately 0.3–1.0 s after the onset of locomotion in the cortex (Figure 3D) and hippocampus (Figure 3F). The mean values of Ca^2+^ transient latencies were 0.81 s in the cortex and 0.66 s in the hippocampus. These data indicated that the locomotion-induced astrocytic Ca^2+^ transients shown by previous two-photon imaging studies (Nimmerjahn et al., 2009; Paukert et al., 2014) can also be recorded by optic fibers in freely behaving mice.

### Footshock-Evoked Astrocytic Ca^2+^ Transients in the Cortices and Hippocampi of Freely Behaving Mice

**Figure 4:**
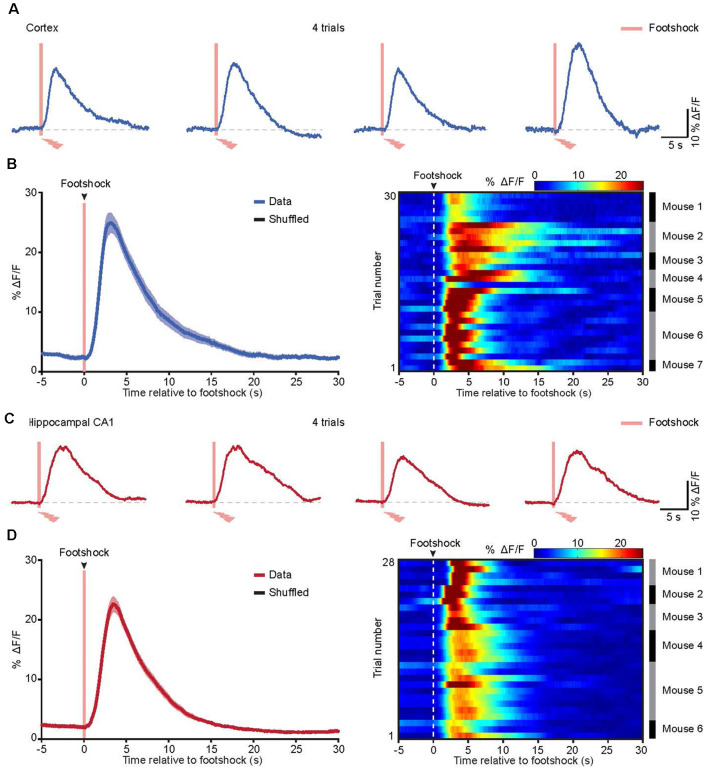
Astrocytic Ca^2+^ transients evoked by footshock in the cortices and hippocampi of freely behaving mice. **(A)** Representative traces showing the astrocytic Ca^2+^ transients evoked by the footshock stimuli (red bars) in the cortex. **(B)** Left, the average (blue trace) of astrocytic Ca^2+^ transients aligned to footshock stimulation (red bar) in the cortices. The average trace was from 30 trials (seven mice). The shaded area represents SEM. Right, color-coded intensities of astrocytic Ca^2+^ transients from different trials in the cortices. The mice which the trials of astrocytic Ca^2+^ transients are from are indicated in the right black and gray column. **(C)** Representative traces showing the astrocytic Ca^2+^ transients evoked by the footshock stimuli (red bars) in the hippocampus.** (D)** Left, the average (red trace) of astrocytic Ca^2+^ transients aligned to footshock stimulation (red bar) in the hippocampi. The average trace was from 28 trials (six mice). The shaded area represents SEM. Right, color-coded intensities of astrocytic Ca^2+^ transients from different trials in the hippocampi. The mice which the trials of astrocytic Ca^2+^ transients are from are indicated in the right black and gray column.

In a previous study, we applied *in vivo* two-photon imaging in head-strained mice and demonstrated that cortical astrocytes could be activated by footshock (Zhang et al., 2016; Zhang and Chen, 2017). To assess the astrocytic Ca^2+^ transients evoked by footshock in the cortices and hippocampi of freely behaving mice, we used an optic fiber-based approach to record astrocytic Ca^2+^ transients in these two brain regions upon application of the footshock stimuli, revealing reliable footshock-evoked astrocytic Ca^2+^ transients in both the cortex (Figure 4A) and the hippocampus (Figure 4C). Further analysis indicated that most of the astrocytic Ca^2+^ transients started approximately 0.3–1.0 s after the onset of footshock stimuli in the cortex (Figure 4B) and hippocampus (Figure 4D). The mean values of Ca^2+^ transient latencies were 0.67 s in the cortex and 0.72 s in the hippocampus. These results indicated that footshock-evoked astrocytic Ca^2+^ transients can also be reliably recorded by optic fibers in freely behaving mice.

### Chronic Recordings of Astrocytic Ca^2+^ Transients in the Brains of Freely Behaving Mice

**Figure 5:**
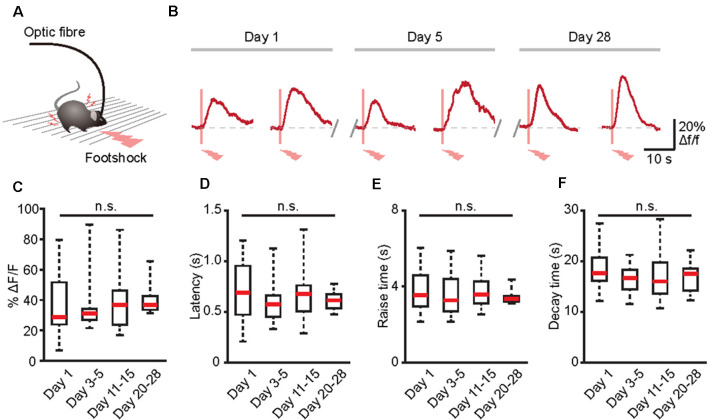
Long-term recording of astrocytic Ca^2+^ transients evoked by footshock stimuli in the cortices of freely behaving mice. **(A)** Schematic of the optic fiber recording in the freely behaving mouse. **(B)** Footshock-evoked astrocytic Ca^2+^ transients in the same mouse on days 1, 5, and 28. **(C)** Summary of the amplitudes of astrocytic Ca^2+^ transients in response to footshock from day 1 to days 20–28. **(D)** Summary of the latencies of astrocytic Ca^2+^ transients in response to footshock from day 1 to days 20–28. **(E)** Summary of the raise time of astrocytic Ca^2+^ transients in response to footshock from day 1 to days 20–28. **(F)** Summary of the decay time of astrocytic Ca^2+^ transients in response to footshock from day 1 to days 20–28. *n* = 10–25 trials from five mice **(C–F)**. Kruskal–Wallis test; n.s., no significant difference.

Many physiological or pathological processes, such as neuromodulation, neural development, and learning/memory, are placed on a long-term axis (Aramuni and Griesbeck, 2013). Thus, to fully understand these processes, data must be extrapolated in the same cells during numerous experimental sessions. Because GECIs may allow the establishment of long-term functional biographies of identified cells (Aramuni and Griesbeck, 2013; Hainmueller and Bartos, 2018), we next combined optic fiber-based recording and GECIs to record the Ca^2+^ transients of the same population of astrocytes in the brains of freely behaving mice for several weeks (Figure 5A). Astrocytic Ca^2+^ transients induced by footshock can be stably recorded for at least 28 days (Figure 5B). Also, there was no difference between the amplitudes, latencies, rise time, and decay time of astrocytic Ca^2+^ transients in response to footshock on different recording days (Figures 5C–F). Therefore, optic fiber-based recordings combined with GECIs are an ideal method for chronic monitoring of astrocytic Ca^2+^ transients in the brains of freely behaving mice.

## Discussion

In recent decades, extensive *in vivo* studies have focused on the role of astrocyte Ca^2+^ transients in the function of neural circuits (Bazargani and Attwell, 2016; Shigetomi et al., 2016; Zhang and Chen, 2017; Santello et al., 2019; Yu et al., 2020). The most relevant experiments were performed in anesthetized or head-fixed awake mice with two-photon microscopy (Hirase et al., 2004; Bekar et al., 2008; Nimmerjahn et al., 2009; Ding et al., 2013; Paukert et al., 2014; Zhang et al., 2016; Oe et al., 2020) and camera-based large-field imaging (Ghosh et al., 2013; Daniel et al., 2015). However, increasing evidence supports that astrocytic Ca^2+^ transients are affected by anesthesia (Nimmerjahn et al., 2009; Thrane et al., 2012) and restraint-induced stress (Oliveira et al., 2015; Oe et al., 2020). Here, we combined optic fiber-based recordings and GECIs to monitor astrocyte Ca^2+^ activities in the brains of freely behaving mice. We have shown that both locomotion and footshock-evoked astrocytic Ca^2+^ transients can be recorded in not only the cortical area but also the subcortical area (hippocampus) of freely behaving mice by optic fibers. More importantly, combined with GECIs, we applied optic fibers to monitor the Ca^2+^ transients of the same population of astrocytes in the brains of freely behaving mice for several weeks. Hence, the combination of optic fiber-based recordings and GECIs provides us with an ideal approach to explore the roles of astrocytic Ca^2+^ transients in the behaviors and cognition functions of freely behaving mice.

Further dissection of the astrocytic Ca^2+^ transients will necessitate simultaneously manipulating and recording astrocytic activities. Recently, we introduced a new variant of fiber photometry that simultaneously enables the optogenetic manipulation and real-time recording of Ca^2+^ activities in the same population of cells (Li et al., 2017; Qin et al., 2018). At the same time, optogenetic tools have been developed for selectively stimulating or attenuating astrocyte Ca^2+^ activities *in vivo* (Yu et al., 2020), including the light-gated ionotropic glutamate receptor (LiGluR), Channelrhodopsin 2 (ChR2), Melanopsin, and opto-XRs (Yu et al., 2020). Therefore, the combination of these optogenetic tools and optic fiber-based recordings will provide opportunities to simultaneously and selectively control and monitor astrocytic activities in freely behaving mice, which will support further analysis of the roles of astrocytic activities in physiological and pathological processes.

Astrocytes represent a diverse population of cells and display brain area-specific properties (Bayraktar et al., 2014; Chai et al., 2017; Khakh and Deneen, 2019). One recent *in vitro* study indicated significant differences between astrocytes located in different brain regions in regards to their electrophysiological properties, Ca^2+^ signaling, morphology, and astrocyte-synapse proximity (Chai et al., 2017). These differences indicate that astrocytic Ca^2+^ monitoring may be a valuable metric for measuring astrocyte diversity at the functional level. However, until now, no *in vivo* evidence has shown whether astrocytes within different brain neural circuits are largely distinct. In recent years, our group and others have presented multichannel fibers enabling photometry from several to tens of brain regions simultaneously (Guo et al., 2015; Kim et al., 2016; Qin et al., 2019; Sych et al., 2019). More importantly, this approach enables simultaneous multichannel fiber photometry and optogenetic perturbations across many brain regions (Sych et al., 2019). Therefore, multichannel fiber photometry offers opportunities for characterizing astrocytic activities and investigating the diversity of astrocytic functions in different brain regions during behavioral performances. Also, although fiber implantation may activate local astrocytes (Karve et al., 2016), these cells are limited in the superficial area around the end of the optic fiber. Most astrocytes in the deep layers show healthy morphology (Figures 3A,B). Because the optical fiber-based system is collecting an average fluorescence signal from superficial as well as from deeper layers (Grienberger et al., 2012), this technique is an appropriate *in vivo* approach to monitoring astrocytic signals.

Although it lacks cellular resolution, the optic fiber has attracted considerable attention because of its simplicity and versatility. In the present study, we demonstrate that the combination of optic fiber recordings and GECIs not only allows the selective monitoring of astrocytic activities but can also be applied to study astrocytic activities for long periods in freely behaving animals. In the future, with the help of optogenetic tools and other sophisticated techniques, we anticipate that optic fiber recordings will become more versatile for investigating the complex functions of astrocytes in the mammalian brain under both health and disease conditions.

## Data Availability Statement

The raw data supporting the conclusions of this article will be made available by the authors, without undue reservation.

## Ethics Statement

The animal study was reviewed and approved by the Institutional Animal Care and Use Committee of Third Military Medical University.

## Author Contributions

HQ, XC, and KZ contributed to the design of the study and interpretation of the data. WH, CY, JL, and TJ carried out the experiments and acquired the data. HQ, WH, CY, JL, TJ, SL, TC, HF, XL, and KZ processed and analyzed the data. XL and KZ wrote the manuscript with help from all other authors. All authors contributed to the article and approved the submitted version.

## Conflict of Interest

The authors declare that the research was conducted in the absence of any commercial or financial relationships that could be construed as a potential conflict of interest.
